# Retraction: Biodegradation of Endocrine-Disrupting Chemicals and Residual Organic Pollutants of Pulp and Paper Mill Effluent by Biostimulation

**DOI:** 10.3389/fmicb.2021.708133

**Published:** 2021-05-31

**Authors:** Frontiers Editorial Office

The journal retracts the May 15, 2018 article cited above.

Following publication, concerns were raised regarding misrepresentation of data and duplication of previously published data. The authors failed to provide a satisfactory explanation during the investigation, which was conducted in accordance with Frontiers' guidelines.

This retraction was approved by the Chief Editors of Frontiers in Microbiology and the Editor-in-Chief of Frontiers. The authors consider the retraction to be unwarranted and do not agree to the statement.

